# Intestinal Sand

**Published:** 1901-04-27

**Authors:** 


### INTESTINAL SAND.

A good deal of discussion has taken place of late as to the nature and meaning ot intestinal sand, the sandy residue which is found after a thorough washing and lixiviation of some specimens of fæces. In a paper read before the Royal Medical and Chirurgical Society, Sir Dyce Duckworth and Dr. A. E. Garrod described a case in which large quantities of this sand had been passed. The patient was a woman, aged 33, in whom the only sign of organic disease was some thickening of the wall of the large intestine, but who had suffered from intractable diarrhœa for two months before the sand was discovered in her motions. This sand was found to be of a reddish-brown colour and gritty, somewhat resembling a uric-acid deposit. It was insoluble in cold and in boiling liquor potassæ, but was readily soluble in boiling nitric acid. Chemical analysis showed it to consist of calcium oxide (about 55 per cent.), phosphorus pentoxide (about 42 per cent.), carbon dioxide (about 2 per cent.) and traces of magnesium and iron. Calcium phosphate was clearly the chief mineral constituent. In the discussion on this paper it was stated that cases of intestinal sand were commoner in India than in this country, but it has since been suggested that this may be because diarrhœa is commoner in India, and thus the sand is more readily discovered. Since then a good deal of correspondence has taken place on the subject, tending to show that the presence of sand in the fæces is a commoner thing than has been generally supposed, and that if looked for it would be often found. It seems clear that in some few cases this so-called sand consists of very minute biliary calculi. But that is not usually the case, as is shown by the results of their analysis. In Sir Dyce Duckworth's case the sandy granules were embedded in an organic basis containing large numbers of bacilli and bacteria. In a case since reported by Dr. Mahomed, the patient passed a quantity of small granules of a pinkish-red colour, which were generally wrapped up in balls of mucus. Although it is possible, as is suggested by some, that we do in fact eat a good deal more dirt of a sandy nature than we are at all aware of, it would seem probable that intestinal sand, properly so-called, and occurring in the quantity in which it is sometimes met with, is in no way due to the swallowing of sandy particles but is the result of some disordered microbial action going on in the intestines, analogous perhaps to those unknown processes, whatever their nature may be, which give rise to the deposit of calculi in other places and from other solutions.

